# Advances in Mechanism of Action and Efficacy of CBP/p300 Inhibitors in Different Subtypes of Breast Cancer

**DOI:** 10.3390/molecules31142426

**Published:** 2026-07-10

**Authors:** Yue Yang, Ting Yang, Yan Lin, Lin Gan

**Affiliations:** 1 Institute for Biochemistry and Molecular Biology, School of Basic Medical Sciences, Southwest Medical University, No. 1 Section 1, Xiang Lin Road, Longmatan District, Luzhou 646000, China; 20240199120038@stu.swmu.edu.cn; 2 Institute for Cancer Medicine, School of Basic Medical Sciences, Southwest Medical University, No. 1 Section 1, Xiang Lin Road, Longmatan District, Luzhou 646000, China; 20240199120037@stu.swmu.edu.cn

**Keywords:** breast cancer, CBP/p300, EP300, epigenetic therapy, biomarker-guided therapy, precision oncology

## Abstract

Breast cancer is a highly heterogeneous malignancy with multiple molecular subtypes and variable treatment responses. Despite advances in endocrine therapy, HER2-targeted therapy, chemotherapy, and immunotherapy, treatment resistance and disease recurrence remain major clinical challenges. There is growing evidence that transcriptional plasticity and enhancer relinking contribute to tumor progression and treatment adaptation, highlighting the powerful role of epigenetic regulators. CREB-binding protein (CBP) and E1A-associated protein p300 (EP300) are transcriptional coactivators that regulate breast cancer enhancer activity and lineage-specific gene expression. Emerging research suggests that CBP/p300 is more of a context-dependent vulnerability point than a universal carcinogenic driver. ER-positive tumors exhibit a strong dependence on CBP/p300-mediated transcriptional programs, while the triple-negative breast cancer subgroup, including androgen receptor-positive and immunosuppressive tumors, may rely on CBP/p300-dependent signaling to maintain survival and treatment resistance. This is in contrast to their role in HER2-positive breast cancer. This review summarizes the biological functions of CBP/p300 in breast cancer and discusses subtype-specific vulnerability, biomarker-directed patient stratification, drug resistance mechanisms, rational combination strategies, and current translational challenges, emphasizing the need for precise treatment of breast cancer.

## 1. Introduction

Breast cancer (BC) remains the most commonly diagnosed malignancy among women worldwide and a leading cause of cancer-related mortality [1]. According to global cancer statistics, approximately 2.31 million new BC cases and more than 660,000 related deaths were reported in 2022, accounting for nearly one in eight newly diagnosed cancers globally. Despite major advances in early detection, systemic therapy, and precision medicine, metastatic breast cancer (MBC) remains largely incurable, with a 5-year survival rate below 30% [2]. Moreover, nearly 20–30% of patients with early-stage disease eventually develop recurrence or distant metastasis, highlighting the persistent need for more effective therapeutic strategies [3].

BC is not a single disease, but a highly heterogeneous malignancy characterized by profound interpatient and intratumoral variability. Molecular classification has stratified BC into several clinically relevant subtypes, including luminal A, luminal B, human epidermal growth factor receptor 2 (HER2)-enriched, and triple-negative breast cancer (TNBC), each exhibiting distinct biological behaviors, therapeutic vulnerabilities, and clinical outcomes [4]. Hormone receptor-positive tumors rely predominantly on estrogen receptor (ER)-driven transcriptional programs, while HER2-positive disease is fueled by oncogenic receptor tyrosine kinase signaling; in contrast, TNBC frequently displays aggressive phenotypes and limited therapeutic options due to the absence of actionable receptors [5]. Although subtype-directed therapies—including endocrine therapy, anti-HER2 agents, cyclin-dependent kinase 4/6 (CDK4/6) inhibitors, poly (ADP-ribose) polymerase (PARP) inhibitors, and immune checkpoint blockade—have improved outcomes for selected patients, therapeutic resistance remains nearly universal in advanced disease [6].

Increasing evidence suggests that treatment failure in breast cancer cannot be fully explained by genomic alterations alone [7]. Instead, tumor evolution and drug resistance are increasingly recognized as consequences of transcriptional plasticity, whereby cancer cells dynamically rewire lineage programs, enhancer landscapes, and adaptive signaling networks in response to therapeutic pressure [8,9]. For example, endocrine-resistant ER-positive tumors frequently maintain dependence on estrogen signaling through estrogen receptor alpha gene (ESR1) mutations, enhancer reprogramming, or redistribution of transcriptional coactivators [10,11], while TNBC commonly acquires drug tolerance through epigenetic state transitions and microenvironmental adaptation [12,13]. These observations suggest that the maintenance of oncogenic transcriptional identity may represent a fundamental vulnerability across multiple breast cancer subtypes [14].

At the center of this transcriptional machinery are the paralogous transcriptional coactivators CREB-binding protein (CBP) and E1A-binding protein p300 (EP300), two multifunctional histone acetyltransferases (HATs) that serve as master regulators of enhancer activity and chromatin accessibility [15]. CBP/p300 integrate extracellular signaling with lineage-specific transcriptional programs through dual functions: acting as scaffolding coactivators for transcription factor complexes and catalyzing lysine acetylation on histone and non-histone proteins, particularly H3K27 acetylation (H3K27ac), a hallmark of active enhancers and super-enhancers [16,17]. Through these functions, CBP/p300 orchestrate oncogenic transcriptional networks involving ER, androgen receptor (AR), Wnt/β-catenin, myelocytomatosis oncogene (MYC), and inflammatory signaling pathways, thereby influencing tumor proliferation, metastasis, immune evasion, and therapeutic response [18,19].

Importantly, emerging evidence indicates that CBP/p300 dependency is highly context-dependent rather than universal [20]. ER-positive breast cancer exhibits pronounced reliance on CBP/p300-mediated enhancer maintenance, especially under endocrine-resistant conditions [21]. In subsets of TNBC, CBP/p300 participates in AR-driven transcription, immune suppression mediated by tumor-associated neutrophils, and transcriptional plasticity associated with chemotherapy resistance [22,23]. Furthermore, the development of multiple pharmacological modalities—including bromodomain inhibitors, catalytic HAT inhibitors, degraders, and dual epigenetic inhibitors—has transformed CBP/p300 from a mechanistic concept into a clinically actionable therapeutic target [24].

In this review, we comprehensively summarize the biological functions of CBP/p300 in breast cancer, focusing on enhancer regulation, subtype-specific vulnerabilities, pharmacologic targeting strategies, biomarkers of dependency, resistance mechanisms, and rational therapeutic combinations. We further discuss current translational bottlenecks and propose a biomarker-driven framework for the precision deployment of CBP/p300-targeted therapies in breast cancer.

## 2. CBP/p300 in Breast Cancer Biology: Enhancer Regulation and Signaling Integration

CBP and p300 function as central transcriptional coactivators that integrate enhancer activity with lineage-specific signaling pathways in breast cancer [15,24]. Mechanistically, CBP/p300 are recruited to chromatin through interactions with diverse DNA-binding transcription factors [25], where they act not only as molecular scaffolds linking enhancers to the basal transcriptional machinery but also as lysine acetyltransferases that deposit histone acetylation marks such as H3K27ac [26,27], a hallmark of active enhancers and super-enhancers [20]. Through this dual functionality, CBP/p300 critically regulate chromatin accessibility and enhancer-driven transcriptional output, thereby sustaining oncogenic transcriptional programs and transcriptional addiction in cancer cells [28].

In hormone receptor-positive breast cancer, CBP/p300 serve as indispensable coactivators of nuclear receptor signaling, particularly ER and AR pathways [29]. Upon ligand stimulation, ER and AR are recruited to enhancer regions and form transcriptional complexes with CBP/p300 and steroid receptor coactivator (SRC) family proteins, leading to increased histone acetylation, chromatin remodeling, and activation of genes involved in proliferation and survival [30,31]. Importantly, CBP/p300-mediated acetylation extends beyond histones to non-histone substrates, including ER itself, thereby enhancing its DNA-binding affinity and transcriptional activity [32]. This establishes CBP/p300 as key amplifiers of hormone-driven enhancer programs, particularly in ER-positive and AR-driven breast cancer contexts, where tumor cells exhibit a strong dependency on lineage-specific transcriptional circuitry [33].

Beyond tumor-intrinsic transcriptional regulation, emerging evidence indicates that CBP/p300 also play a critical role in shaping the tumor immune microenvironment. By regulating the transcription of immune-related genes—including cytokines, antigen presentation machinery, and immune checkpoint molecules—CBP/p300 contribute to the dynamic balance between immune activation and immune evasion [22]. Their acetyltransferase activity modulates key inflammatory signaling pathways such as NF-κB, thereby influencing the recruitment and functional polarization of immune cells within the tumor microenvironment [34]. In particular, CBP/p300-dependent transcriptional programs in myeloid cells and tumor-associated neutrophils may further reinforce immunosuppressive states in certain breast cancer contexts [22].

**Figure 1:**
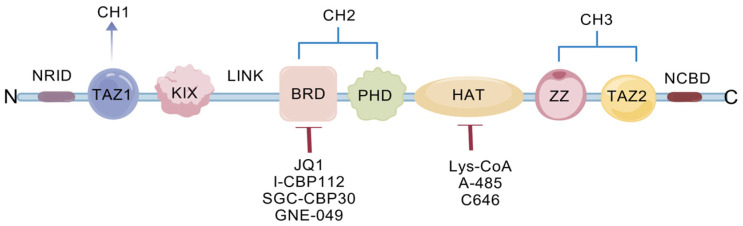
The structure of CBP/P300. CBP/p300 protein domains (from N-terminus to C-terminus): nuclear receptor interaction domain (NRID): mediates binding to nuclear receptors; transcriptional adapter zinc finger domain 1 (TAZ1): binds to transcription factors; CREB interaction domain (KIX): inducible by kinases, involved in signal transduction; bromodomain (BRD): recognizes acetylated lysine, participates in chromatin remodeling; plant homeodomain (PHD): recognizes methylated or acetylated histones; histone acetyltransferase catalytic core domain (HAT); ZZ-type zinc finger domain (ZZ): potentially involved in protein–protein interactions; transcriptional adapter zinc finger domain 2 (TAZ2); nuclear receptor coactivator binding domain (NCBD). Inhibitors of the CBP/P300 BRD include JQ1, ICBP112, SCG-CBP30, and GNE-049. Inhibitors of the CBP/P300 HAT domain include Lys-CoA, A-485, and C646.

Collectively, CBP/p300 act as central hubs that couple enhancer activity with hormone receptor signaling and immune regulation. Importantly, this integration is highly context-dependent, providing a mechanistic basis for the differential transcriptional dependencies observed across breast cancer subtypes and, consequently, their distinct vulnerabilities to CBP/p300-targeted therapies (Figure 1).

## 3. Pharmacological Strategies Targeting CBP/p300: Modality-Specific Transcriptional Control

CBP/p300 targeting has evolved from early non-selective HAT inhibitors to a diverse set of mechanistically distinct pharmacological strategies, including bromodomain (BRD) inhibitors, catalytic HAT inhibitors, proteolysis-targeting chimeras (PROTACs), and dual-target inhibitors. Rather than representing merely different classes of compounds, these modalities impose distinct modes of transcriptional regulation that vary in their selectivity, magnitude, and durability of effect. As such, they should be understood as functionally non-equivalent tools that differentially perturb enhancer-driven transcriptional programs across breast cancer contexts.

### 3.1. BRD Inhibitors: Selective Interference with Enhancers

The BRD of CBP/p300 acts as a reader module that recognizes acetylated lysine residues on histone and non-histone proteins, thereby facilitating chromatin recruitment and stabilizing transcriptional complexes [35,36]. Pharmacological inhibition of this domain, exemplified by compounds such as SGC-CBP30 [37], GNE-049 [38], and I-CBP112 [39], disrupts the interaction between CBP/p300 and acetylated substrates, leading to impaired localization in active chromatin regions. Notably, BRD inhibition preferentially affects enhancer-associated H3K27ac rather than promoter regions, reflecting the greater dependence of enhancers—particularly super-enhancers—on sustained CBP/p300 engagement [17,28]. This “enhancer bias” results in selective attenuation of enhancer-driven transcriptional outputs without inducing a global shutdown of gene expression. Such a property is particularly relevant in breast cancer subtypes characterized by enhancer addiction [40], including ER-positive tumors in which forkhead box protein A1/GATA binding protein 3 (FOXA1/GATA3) dependent enhancer networks sustain lineage-specific transcriptional programs [41]. In addition, BRD inhibition has been reported to interfere with CBP/p300 recruitment to DNA damage sites, thereby impacting DNA repair processes [42].

### 3.2. HAT Inhibitors: Global Catalytic Inhibition and Transcriptional Repression

In contrast to BRD inhibitors, HAT inhibitors directly target the catalytic activity of CBP/p300 by competing with acetyl-CoA binding, resulting in a global reduction in lysine acetylation across both histone and non-histone substrates [43]. This includes key regulatory marks such as H3K27ac and histone H3 lysine 18 acetylation (H3K18ac), as well as transcription factors including tumor protein p53 (p53) and ERα [44]. Reduced acetylation of p53 may impair its transcriptional activity and consequently attenuate p53-mediated cell-cycle arrest and apoptosis in tumors retaining wild-type gene encoding p53 (TP53) [45]. However, because TP53 is frequently mutated in breast cancer, especially in triple-negative breast cancer, the therapeutic effects of CBP/p300 inhibitors are considered to primarily result from suppression of enhancer-dependent oncogenic transcription rather than modulation of p53 signaling [17,21]. Consequently, HAT inhibition leads to a more profound suppression of transcriptional activity compared with BRD inhibition, effectively collapsing acetylation-dependent oncogenic transcriptional programs [46]. However, this broader mode of action is accompanied by reduced selectivity and a higher likelihood of affecting normal cellular processes [20]. The highly selective HAT inhibitor A-485 has demonstrated potent suppression of ER target gene expression and induction of cellular senescence in ER-positive breast cancer models [33], while other inhibitors such as L002 have shown antitumor activity in triple-negative breast cancer models [47]. These observations suggest that HAT inhibition may be particularly suited for tumor contexts that require deep transcriptional repression, such as endocrine-resistant disease or highly transcriptionally active malignancies.

### 3.3. PROTAC Degradation Agents: Complete Elimination vs. Functional Inhibition

More recently, PROTAC-based strategies have introduced the possibility of directly eliminating CBP/p300 proteins rather than merely inhibiting their activity [48,49]. By recruiting E3 ubiquitin ligases such as cereblon (CRBN) or RNF126, CBP/p300 degraders induce ubiquitination and subsequent proteasomal degradation of the target proteins [50,51]. This approach offers the conceptual advantage of removing both the catalytic and non-catalytic functions of CBP/p300, thereby overcoming potential limitations associated with partial inhibition [49]. In addition, protein degradation may mitigate resistance mechanisms arising from mutations that impair inhibitor binding while preserving protein function [52]. Preclinical studies have demonstrated that degraders such as dCBP-1 can suppress MYC-driven enhancer activity, and emerging molecules such as A8 have shown antiproliferative effects in breast cancer cell lines. Nevertheless, challenges related to molecular size, pharmacokinetic properties, and potential toxicity due to the depletion of CBP/p300 in normal tissues remain significant barriers to clinical translation [50,51].

### 3.4. Dual-Target Inhibitors: BET/CBP-p300 Synergistic Strategy

In parallel, dual-target inhibitors have been developed to simultaneously disrupt CBP/p300 and bromodomain and extra-terminal domain family (BET) proteins, particularly bromodomain-containing protein 4 (BRD4), which cooperatively regulate super-enhancer-driven transcription [53]. Compounds such as NEO2734 and NEO1132 are designed to block both the “writing” of acetylation marks by CBP/p300 and the “reading” of these marks by BET proteins, thereby exerting more comprehensive suppression of transcriptional circuitry [54,55]. Preclinical evidence from hematologic malignancies and prostate cancer models suggests enhanced efficacy compared with single-agent approaches, although their application in breast cancer remains relatively underexplored [56]. Importantly, the simultaneous targeting of multiple epigenetic nodes may also increase the risk of toxicity, highlighting the need for careful therapeutic optimization [57].

Taken together, these pharmacological strategies can be conceptualized along a continuum of transcriptional perturbation, ranging from selective modulation of enhancer activity to global suppression or complete elimination of CBP/p300 function [23,53]. Importantly, this spectrum underscores that no single modality is universally optimal; rather, the therapeutic effectiveness of CBP/p300 targeting is likely to depend on the specific transcriptional dependencies of each tumor context [29]. Breast cancers driven by enhancer-centric programs may preferentially respond to BRD inhibition, whereas tumors with more aggressive or resistant phenotypes may require deeper transcriptional disruption through catalytic inhibition or targeted degradation. This perspective highlights the importance of aligning the pharmacological strategy with the tumor biology and supports the development of biomarker-guided approaches for the clinical deployment of CBP/p300-targeted therapies.

### 3.5. Advantages and Disadvantages of Different Modalities and Their Applicable Scenarios

To provide clearer guidance for selecting CBP/p300 targeting strategies across different breast cancer subtypes, we summarize the core characteristics, advantages, limitations, and potential application scenarios of various pharmacological approaches in Table 1.

### 3.6. Safety Profile and Adverse Effects of CBP/p300 Inhibitors

Although CBP/p300 inhibitors have demonstrated encouraging antitumor activity in preclinical models, their safety profile remains an important concern for clinical translation. Because CBP and p300 are ubiquitously expressed transcriptional coactivators involved in normal tissue homeostasis, their systemic inhibition may affect multiple physiological processes beyond tumor cells.

The toxicity profiles vary substantially among different classes of CBP/p300 inhibitors [23]. BRD inhibitors generally exhibit relatively favorable tolerability because they selectively interfere with acetyl-lysine recognition while largely preserving the catalytic acetyltransferase activity of CBP/p300 [25,58]. Consequently, these compounds primarily affect enhancer-dependent transcription and are expected to induce fewer systemic adverse effects than catalytic inhibitors.

In contrast, HAT inhibitors suppress the acetyltransferase activity of CBP/p300 globally, resulting in widespread reductions in histone and non-histone protein acetylation [23,59]. Such broad inhibition may impair normal cellular transcription, leading to dose-limiting toxicities, including hematopoietic suppression, gastrointestinal toxicity, and hepatic dysfunction [60]. In addition, inhibition of p53 acetylation may potentially compromise normal stress responses and tissue repair [45].

PROTAC degraders may further increase toxicity by completely eliminating CBP/p300 proteins, thereby abolishing both catalytic and scaffolding functions [61]. While this strategy may overcome resistance associated with incomplete target inhibition, prolonged depletion of CBP/p300 in normal tissues raises concerns regarding developmental, immune, and metabolic toxicity [62,63,64].

Clinical experience with currently available CBP/p300 inhibitors remains limited but encouraging [20]. Early-phase studies of CCS1477, FT-7051, and TT125-802 have generally demonstrated manageable safety profiles, with fatigue, nausea, decreased appetite, anemia, and thrombocytopenia being among the most frequently reported treatment-related adverse events [19]. Notably, TT125-802 showed preliminary antitumor activity without inducing significant thrombocytopenia, suggesting that improvements in compound selectivity may widen the therapeutic window [65]. Nevertheless, long-term toxicity data and breast cancer-specific safety evidence remain scarce, emphasizing the need for further clinical evaluation.

## 4. Subtype-Specific Vulnerabilities and Therapeutic Rationales

### 4.1. ER-Positive Breast Cancer: Enhancer-Driven Lineage Dependency

ER-positive breast cancer represents the most well-defined context of CBP/p300 dependency, which is fundamentally rooted in enhancer-driven lineage-specific transcriptional programs [21,66]. The growth and survival of these tumors rely on ERα-mediated transcription, which is orchestrated through a highly organized enhancer landscape [33,67]. Within this framework, CBP/p300 are recruited to ER-bound regulatory regions, where they deposit H3K27ac to maintain chromatin accessibility and sustain transcriptional output. Genome-wide studies have demonstrated extensive co-occupancy of ERα and CBP/p300, highlighting their integral role in enhancer function [31]. This process is further facilitated by pioneer factors such as FOXA1 and GATA3, which establish accessible chromatin regions and enable ER binding to noncanonical sites [68]. CBP/p300 then reinforce these regions through acetylation, forming a hierarchical cascade of enhancer activation that ultimately drives oncogenic transcriptional programs [38,69]. Consequently, ER^+^ breast cancer exhibits a form of enhancer dependency that renders it particularly sensitive to perturbations in CBP/p300 activity.

Importantly, this dependency is largely preserved in the context of endocrine resistance [70,71]. Although resistance to therapies such as tamoxifen or aromatase inhibitors frequently arises, many resistant tumors remain reliant on ER signaling through mechanisms including ESR1-activating mutations, enhancer reprogramming, and increased coactivator activity. Functional studies have demonstrated that CBP/p300 remain essential for the proliferation of ER-mutant cancer cells, indicating that these coactivators operate downstream of ligand binding and are not bypassed by classical resistance pathways [72]. Pharmacological inhibition of CBP/p300 disrupts enhancer-associated acetylation and suppresses ER target gene expression even in resistant settings, thereby providing an orthogonal therapeutic strategy that targets the transcriptional machinery rather than receptor activation itself [73]. In this context, different pharmacological modalities may offer distinct advantages: catalytic inhibition can induce deep suppression of transcriptional output, whereas BRD inhibition preferentially attenuates enhancer activity with potentially improved selectivity and tolerability. Together, these features position ER-positive breast cancer as a prototypical model of lineage-driven CBP/p300 dependency and a primary candidate for therapeutic intervention.

### 4.2. TNBC: Heterogeneous and Multi-Axis CBP/p300 Dependency

TNBC is a highly heterogeneous disease in which CBP/p300 dependency does not arise from a single dominant pathway but instead reflects multiple context-specific transcriptional programs [74]. These dependencies can be broadly categorized into nuclear receptor coactivation, immune microenvironment regulation, and resistance-associated transcriptional plasticity, each defining a distinct therapeutic entry point for CBP/p300-targeted strategies.

#### 4.2.1. AR-Positive/LAR-TNBC: Nuclear Receptor Coactivation Dependency

A subset of TNBC, commonly referred to as the luminal androgen receptor (LAR) subtype, is characterized by AR expression and reliance on AR-driven transcriptional programs [75]. In this context, CBP/p300 function as critical coactivators of AR, analogously to their role in ER-positive breast cancer. Upon ligand activation, AR is recruited to enhancer regions where it forms transcriptional complexes with CBP/p300, leading to histone acetylation, chromatin remodeling, and activation of genes involved in proliferation and survival. This establishes a form of nuclear receptor coactivation dependency in which tumor growth is sustained by CBP/p300-mediated amplification of AR signaling. Pharmacological inhibition of CBP/p300, particularly through bromodomain-targeting compounds, disrupts AR-dependent transcription and selectively suppresses proliferation in AR-positive TNBC models while sparing AR-negative cells [21,29]. These findings suggest that CBP/p300 inhibition may serve as an alternative or complementary strategy to anti-androgen therapies, particularly in tumors that exhibit incomplete or adaptive resistance to direct AR blockade.

#### 4.2.2. Neutrophil-Enriched/Immune-Suppressive TNBC: Microenvironmental Dependency and Immunomodulation

In another subset of TNBC, tumor progression is strongly influenced by the immune microenvironment, particularly in tumors characterized by high infiltration of tumor-associated neutrophils (TANs) and other myeloid-derived suppressor populations [76,77,78]. In these settings, CBP/p300 regulate transcriptional programs that sustain immunosuppressive phenotypes, including the expression of cytokines, chemokines, and immune checkpoint-related molecules. By maintaining these gene expression programs [79,80], CBP/p300 contribute to the exclusion or dysfunction of cytotoxic T-cells and the establishment of an immune “cold” tumor state [17]. Pharmacological inhibition of CBP/p300 has been shown to disrupt these transcriptional networks, leading to reduced recruitment and suppressive activity of TANs, enhanced CD8^+^ T-cell infiltration, and increased interferon signaling within the tumor microenvironment [22]. This reprogramming effect suggests that CBP/p300 inhibitors may function as epigenetic immune modulators capable of converting immunologically inert tumors into responsive ones [81]. Consequently, combining CBP/p300 inhibition with immune checkpoint blockade, such as anti-programmed death-1 (anti-PD-1) or anti-PD-L1 therapy, represents a rational strategy to enhance therapeutic efficacy in this subset of TNBC.

#### 4.2.3. Therapy-Resistant TNBC: Transcriptional Plasticity and Drug Resistance Dependency

Therapy resistance represents a major clinical challenge in TNBC and is frequently associated with transcriptional plasticity that enables tumor cells to adapt to therapeutic pressure [82,83]. CBP/p300 play a central role in this process by maintaining enhancer activity for genes involved in drug efflux, stress response, and survival pathways. In particular, ATP-binding cassette (ABC) transporters such as ATP-binding cassette subfamily B member 1 (ABCB1) and ATP-binding cassette subfamily G member 2 (ABCG2) are transcriptionally regulated in a CBP/p300-dependent manner, contributing to multidrug resistance by reducing intracellular drug accumulation. Inhibition of CBP/p300 disrupts enhancer-associated acetylation at these loci, leading to decreased expression of drug efflux transporters and restoration of chemotherapy sensitivity [84,85,86]. Beyond specific resistance genes, CBP/p300 also support broader transcriptional reprogramming that allows tumor cells to transition between cellular states, thereby facilitating escape from targeted therapies [87]. By constraining this adaptive transcriptional flexibility, CBP/p300 inhibition may limit the emergence of resistant clones and prolong therapeutic response. These findings highlight CBP/p300 as key regulators of resistance-associated transcriptional plasticity and support their use in combination with conventional chemotherapy or targeted agents to overcome or delay resistance in TNBC [88].

### 4.3. HER2-Positive Breast Cancer: Limited Evidence and Context-Dependent Potential

Compared with that in the ER-positive and TNBC subtypes, the role of CBP/p300 in HER2-positive breast cancer remains less clearly defined, and the relative paucity of evidence highlights an important gap in current understanding [89]. Available data do not support a universal dependency of HER2-driven tumors on CBP/p300-mediated transcription and, in some experimental systems, sensitivity to CBP/p300 inhibition appears to be driven by coexisting androgen receptor activity rather than HER2 signaling itself. Nevertheless, several lines of indirect evidence suggest that CBP/p300 may still contribute to tumor progression and therapeutic resistance in specific contexts. HER2 signaling activates downstream pathways such as MAPK and PI3K/AKT, which can influence chromatin dynamics and enhancer activity, potentially modulating CBP/p300 function [90,91,92]. Furthermore, resistance to anti-HER2 therapies is frequently associated with transcriptional reprogramming and phenotypic plasticity, processes that are likely to involve enhancer remodeling and may therefore engage CBP/p300-dependent mechanisms. The involvement of CBP-associated β-catenin signaling in HER2-driven tumorigenesis further supports a possible role for CBP/p300 in this setting, albeit indirectly [93,94,95].

Taken together, these findings suggest that CBP/p300 targeting in HER2-positive breast cancer is unlikely to represent a broadly applicable monotherapy strategy but may instead hold value in specific biological contexts, such as therapy resistance, pathway crosstalk, or particular molecular subgroups. This context-dependent perspective underscores the need for more systematic investigation into the role of CBP/p300 in HER2-driven disease and highlights the importance of biomarker-guided approaches in future translational studies.

## 5. Biomarkers, Resistance, and Rational Combinations

### 5.1. Biomarker Framework for Predicting CBP/p300 Dependency

Precise targeting of CBP/p300 requires not only an understanding of subtype-specific dependencies but also the establishment of a multi-layered biomarker framework that captures tumor-intrinsic features, enhancer landscape activity, and microenvironmental context [96,97,98]. Rather than relying on single markers, effective patient stratification will likely depend on integrating these dimensions to define CBP/p300-dependent transcriptional states [99,100].

#### 5.1.1. Tumor-Intrinsic Biomarkers

Tumor-intrinsic features represent the most accessible layer for initial patient stratification [101]. Expression of nuclear receptors such as ER and AR serves as a primary indicator of CBP/p300 dependency, reflecting reliance on lineage-specific transcriptional programs [102]. Waddell et al. reported that pharmacologic inhibition of CBP/p300 with A-485 or GNE-049 markedly reduced H3K27ac deposition at ER-associated enhancers and suppressed canonical ER target genes, including MYC and Cyclin D1, even in endocrine-resistant settings [33]. Preclinical studies demonstrated that the bromodomain inhibitor FT-6876 preferentially inhibited proliferation in MDA-MB-453 cells, a canonical AR-positive TNBC model, while exerting substantially weaker effects in AR-negative TNBC lines [103]. Beyond receptor expression, co-expression of pioneer factors such as FOXA1 and GATA3 further amplifies enhancer-driven transcriptional programs and may indicate heightened CBP/p300 dependency [67]. In clinical practice, these features can be assessed using immunohistochemistry or transcriptomic profiling and serve as a practical first-tier screening strategy [104].

#### 5.1.2. Enhancer-State Biomarkers

At a mechanistic level, CBP/p300 dependency is closely linked to enhancer activity, making epigenomic features a critical second layer of biomarkers [96]. H3K27ac, a direct product of CBP/p300 catalytic activity, serves as a surrogate marker of active enhancers and super-enhancers [105]. Tumors with elevated global H3K27ac levels or expanded super-enhancer landscapes are more likely to exhibit transcriptional addiction to CBP/p300 [106,107]. Tumors dominated by FOXA1/ER super-enhancer circuitry may preferentially respond to bromodomain inhibitors such as GNE-049 [33], which selectively disrupt enhancer occupancy without globally abolishing acetylation. In contrast, tumors exhibiting widespread chromatin rewiring and diffuse H3K27ac activation may require catalytic HAT inhibition using agents such as A-485 [108]. Technologies such as ChIP-seq or CUT&Tag enable detailed mapping of enhancer landscapes, while immunohistochemical detection of H3K27ac may provide a clinically feasible approximation [109,110]. Importantly, the degree of enhancer dependency may also inform modality selection: tumors with highly enhancer-centric transcriptional programs may preferentially respond to bromodomain inhibitors, which selectively disrupt enhancer activity, whereas broader acetylation dependence may necessitate catalytic inhibition [18,23].

#### 5.1.3. Microenvironmental Biomarkers

In TNBC, microenvironmental features represent a third critical layer of stratification [111,112]. Tumors enriched with tumor-associated neutrophils or myeloid-derived suppressor cells exhibit CBP/p300-dependent immunosuppressive transcriptional programs [22,113]. High neutrophil infiltration, myeloid gene signatures, and low baseline interferon signaling may predict responsiveness to CBP/p300 inhibition, particularly in combination with immune checkpoint blockade [114,115]. Yuan et al. demonstrated that the CBP/p300 bromodomain inhibitor IACS-70654 reduced TAN infiltration in TNBC mouse models, accompanied by increased CD8^+^ T-cell accumulation and improved response to anti-PD-1 therapy [22].

Moreover, CBP/p300 inhibition has been shown to enhance antigen presentation and interferon responses, suggesting that tumors with low baseline immunogenicity may derive the greatest benefit [116,117,118]. These features can be assessed through multiplex immunohistochemistry, flow cytometry, or transcriptomic scoring systems, enabling integration of the immune context into patient selection [119,120].

### 5.2. Mechanisms of Resistance to CBP/p300 Inhibition

Despite promising preclinical activity, resistance to CBP/p300-targeted therapies is likely to emerge through adaptive rewiring of transcriptional and signaling networks [121]. A central theme underlying these mechanisms is the ability of tumor cells to restore transcriptional output through alternative pathways [86,122].

#### 5.2.1. Activation of Bypass Signaling Pathways

One of the most direct adaptive responses to CBP/p300 inhibition is the compensatory activation of parallel signaling pathways, which maintain cell proliferation independently of enhancer-dependent transcription.

In ER-positive breast cancer, inhibition of CBP/p300-mediated enhancer activity may attenuate ER-driven transcription while inducing compensatory activation of signaling pathways such as MAPK and PI3K/AKT, enabling tumor cells to bypass dependence on enhancer-driven mechanisms [123,124]. In TNBC, excessive activation of the receptor tyrosine kinase pathway or inflammatory signaling circuits may compensate for transcriptional inhibition, enabling tumor cells to maintain growth despite disruption by enhancers [110]. These findings suggest that CBP/p300 inhibition alone may not fully suppress oncogenic signals, supporting the rationale for a vertical combination blockade—pairing CBP/p300 inhibitors with pathway-specific inhibitors targeting PI3K, MEK, or receptor tyrosine kinases. This adaptive signaling pathway reprogramming underscores the importance of a combined strategy targeting both transcriptional mechanisms and upstream oncogenic pathways.

#### 5.2.2. Chromatin Reprogramming and Epigenetic Compensation

In addition to signal adaptation, tumors may also evade CBP/p300 inhibition through direct reorganization of the chromatin structure and epigenetic compensation. Since enhancer activity is maintained through coordinated interactions among various chromatin regulatory factors, activation of other epigenetic programs may prevent CBP/p300-mediated inhibition from completely abolishing transcription.

A potential mechanism involves compensatory upregulation of other histone acetyltransferases, including p300/CBP-associated factor (PCAF) and general control nonderepressible 5 (GCN5), which can partially restore histone acetylation and maintain enhancers. Although these enzymes cannot fully replicate the extensive coactivator function of CBP/p300, their enhanced activity may sustain adequate residual transcription to ensure tumor survival [125,126]. Meanwhile, chromatin remodeling complexes such as switch/sucrose non-fermentable chromatin remodeling complex (SWI/SNF) may reorganize enhancer–promoter interactions, facilitating the emergence of alternative transcriptional programs. Additionally, bromodomain-containing proteins like BRD4 may function as compensatory transcription readers; enhanced recruitment or activity of BRD4 can maintain transcriptional elongation under CBP/p300 inhibition, thereby mitigating the effects of bromodomain inhibitors. This compensatory mechanism highlights the dynamic interactions between acetylation “writers” and “readers,” providing a mechanistic basis for the BET/CBP-p300 dual-directed strategy [127,128].

#### 5.2.3. Wnt/β-Catenin-Mediated Escape Mechanisms

Among the candidate escape pathways, Wnt/β-catenin signaling is particularly relevant. CBP-mediated β-catenin transcriptional activity is implicated in maintaining stem cell identity, promoting tumor progression, and conferring therapeutic resistance [18]. Although inhibitors such as ICG-001 selectively disrupt the CBP–β-catenin interaction, tumors harboring fixed β-catenin activation or stable mutations may exhibit reduced dependence on typical CBP-mediated transcriptional regulation. In such cases, β-catenin-driven transcription may serve as an alternative mechanism to sustain carcinogenic processes despite widespread enhancer suppression [129,130].

#### 5.2.4. Metabolic Adaptation and Acetyl-CoA Availability

Emerging evidence suggests that metabolic rewiring may also contribute to resistance by preserving acetylation potential under catalytic inhibition. Because CBP/p300 enzymatic activity depends on acetyl-CoA availability, cellular metabolism may influence the effectiveness of HAT inhibitors in ways that extend beyond direct target engagement [131,132].

Tumor cells exposed to catalytic CBP/p300 inhibition may compensate by increasing intracellular acetyl-CoA pools through upregulation of metabolic enzymes such as ATP citrate lyase (ACLY) or acetyl-CoA synthetase 2 (ACSS2). Increased substrate availability could sustain residual acetylation activity or enhance the function of compensatory acetyltransferases, thereby partially preserving chromatin accessibility and transcriptional output [133,134]. Additionally, changes in nutrient utilization and the intracellular compartmentalization of acetyl-CoA may further buffer the effects of catalytic inhibition. Although these mechanisms remain incompletely understood, they suggest that the metabolic state may critically shape therapeutic sensitivity and raise the possibility that metabolic co-targeting strategies could restore responsiveness to CBP/p300 inhibitors [121].

### 5.3. Rational Combination Strategies

Given the central role of CBP/p300 in coordinating transcriptional programs, monotherapy is unlikely to achieve durable responses, and rational combination strategies will be essential to enhance efficacy and overcome resistance [20,23]. These combinations should be guided by the principle of targeting complementary nodes within the transcriptional network.

#### 5.3.1. Combination with Endocrine Therapy in ER^+^ Breast Cancer

In ER-positive disease, CBP/p300 inhibitors and endocrine therapies target distinct nodes within the same signaling axis, providing a strong rationale for combination [108]. While endocrine therapies inhibit ligand binding or degrade ERα, CBP/p300 inhibition disrupts enhancer-mediated transcriptional activation, resulting in dual blockade of ER signaling [135].

#### 5.3.2. Combination with Chemotherapy in TNBC

In TNBC, CBP/p300 inhibition can sensitize tumors to chemotherapy by reducing drug efflux, impairing DNA repair, and limiting transcriptional plasticity [136]. This multi-layered effect enhances the cytotoxic impact of conventional agents and may help overcome resistance [137].

#### 5.3.3. Combination with Immunotherapy

CBP/p300 BRD inhibitors can remodel the immunosuppressive microenvironment by reducing TAN infiltration and function while increasing CD8^+^ T-cell infiltration and interferon gamma (IFNγ) production. This provides a strong rationale for their combination with immune checkpoint inhibitors (anti-PD-1/PD-L1). In TNBC mouse models, the combination of a CBP/p300 BRD inhibitor with anti-PD-1 showed synergistic inhibition of tumor growth and prolonged survival [22]. Moreover, CBP/p300 inhibition can upregulate major histocompatibility complex (MHC) class I molecule expression on tumor cells, enhance antigen presentation, and further increase sensitivity to immunotherapy [138]. This “epigenetic immune priming” strategy is particularly applicable to neutrophil-enriched or “cold” tumors that originally respond poorly to immunotherapy [139].

#### 5.3.4. Emerging Combinations with Targeted Therapies

CDK4/6 inhibitors are the standard of care for ER-positive breast cancer [140]. Combining CBP/p300 inhibitors with CDK4/6 inhibitors may synergistically inhibit the cell cycle: CBP/p300 inhibitors downregulate Cyclin D1, while CDK4/6 inhibitors directly block CDK activity [141]. In addition, CBP/p300 is involved in DNA damage repair [142]. PARP inhibitors (PARPis) are effective in BRCA-mutated breast cancer [143]. The concurrent use of CBP/p300 inhibitors may enhance PARPi sensitivity by inhibiting homologous recombination repair and thereby expand their application to breast cancer susceptibility gene (BRCA) wild-type tumors [144]. These combination strategies are currently at an early preclinical exploratory stage but harbor considerable translational potential. We summarize the synergistic strategies combining CBP/P300 inhibitors with other therapeutic approaches in Table 2.

## 6. Clinical Translation Landscape and Bottlenecks

Preclinical studies on CBP/p300 inhibitors have accumulated substantial evidence demonstrating their therapeutic potential across various breast cancer subtypes; however, the translational process from the laboratory to clinical application still faces numerous bottlenecks. The central question in this field is no longer “whether CBP/p300 inhibitors are effective” but, rather, “under what biological conditions, with what strategies, and in which patient populations should they undergo clinical validation?”

### Clinically Approved CBP/p300 Inhibitors and Their Progress Across Pan-Cancer Models

Currently, several CBP/p300-targeting molecules have entered the early clinical trial stage, including the bromodomain inhibitors CCS1477, FT-7051, and TT125-802 [145] and a dual-target inhibitor, NEO2734 (EP31670), targeting both CBP/p300 and BET. The clinical development of these drugs is primarily focused on hematologic malignancies and prostate cancer, with extremely limited data available for breast cancer [65].

CCS1477 was the first oral p300/CBP bromodomain inhibitor to enter clinical trials, targeting the AR and c-Myc signaling pathways [146]. It is currently undergoing Phase II clinical studies for non-Hodgkin lymphoma, and its application has been expanded to solid tumors, including castration-resistant prostate cancer (mCRPC), metastatic breast cancer, and non-small cell lung cancer (NSCLC) [147]. Preclinical studies have also demonstrated that CCS1477 inhibits NRF2-dependent chemotherapy-resistant transcriptional programs and reverses the transcriptional state of T-cell exhaustion, providing a novel rationale for combination chemotherapy and immunotherapy [123].

FT-7051 is also an oral CBP/p300 bromodomain inhibitor that exerts antitumor effects by targeting the AR pathway. The drug has completed Phase I Courage trials (NCT04575766) in patients with mCRPC, demonstrating favorable safety and pharmacokinetic profiles [65]. It has now advanced to Phase 1b/2a trials to investigate monotherapy strategies as well as combination therapies with abiraterone, olaparib, or 177Lu-PSMA-617 [19]. Notably, the investigators emphasized the accompanying biomarker strategy designed to identify the patient populations most likely to benefit.

TT125-802 is another selective CBP/p300 bromodomain inhibitor that has recently garnered attention as the first CBP/p300 inhibitor to demonstrate definitive clinical activity in solid tumors [18]. Its Phase I clinical trial (NCT06403436) enrolled a NSCLC cohort from among patients with extensive prior disease, achieving partial responses in three out of 14 patients [20]. More importantly, the drug did not induce thrombocytopenia—a common adverse effect associated with similar inhibitors—demonstrating an optimal safety profile for its class [148]. This groundbreaking finding opens a translational window for the application of CBP/p300 inhibitors in a broader range of solid tumors, including breast cancer.

NEO2734 (EP31670) is a first-in-class dual-target inhibitor targeting both the CBP/p300 and BET bromodomain domains, demonstrating superior antitumor activity compared to single-target agents in preclinical models of prostate cancer and hematologic malignancies [54]. A Phase I first-in-human study (NCT05488548) is currently underway, primarily evaluating its safety, tolerability, and maximum tolerated dose in patients with mCRPC, nuclear protein in testis (NUT) midline carcinoma, chronic myelomonocytic leukemia (CMML), and other targeted advanced solid tumors [23]. This drug holds promise for delivering a “double-targeting” therapeutic strategy against tumors highly dependent on super-enhancer transcription.

Furthermore, the development of selective CBP degraders and EP300 degraders is advancing rapidly [50]. Foghorn Therapeutics’ selective CBP degrader has entered the non-GLP toxicology research phase, targeting EP300-mutant tumors and ER-positive breast cancer, and is expected to reach investigational new drug (IND) ready status by 2026, providing a new translational opportunity for the application of PROTAC technology in breast cancer treatment [48]. In Table 3, we summarize the significance of clinically validated molecules targeting CBP/p300 in breast cancer.

## 7. Discussion

Although epigenetic strategies targeting CBP/p300 have demonstrated significant antitumor potential in preclinical models, their translation into clinical applications for breast cancer has lagged markedly behind that for AR-driven malignancies such as hematologic tumors and prostate cancer. Most CBP/p300 inhibitors currently entering early-stage clinical trials adhere to a “pathway-specific” development paradigm, primarily leveraging AR signaling-dependent biological mechanisms. While this approach is pragmatic, it overlooks the unique transcriptional dependencies specific to breast cancer. In reality, CBP/p300-dependent mechanisms exhibit fundamental differences across various breast cancer subtypes, yet existing clinical trial designs fail to implement prospective biomarker stratification based on these subtype-specific transcriptional vulnerabilities. This tripartite structural misalignment—between “mechanism verification,” “heterogeneity design,” and “pharmacology endpoint”—creates an important translational gap between preclinical evidence and clinical benefit.

A further challenge lies in the fact that CBP/p300 are ubiquitously expressed transcriptional coactivators that participate in normal physiological gene regulation. Consequently, systemic inhibition may inevitably affect normal tissues and result in on-target toxicity. Nevertheless, early clinical studies have demonstrated that several CBP/p300 inhibitors exhibit manageable safety profiles in patients with advanced solid tumors, suggesting that tumor cells may display a greater degree of “transcriptional addiction” to CBP/p300 than normal tissues, thereby creating a therapeutic window. Importantly, toxicity should be considered in a modality-dependent manner rather than as a uniform limitation of all CBP/p300-targeting agents. BRD inhibitors generally display a more favorable safety profile because they selectively interfere with enhancer-associated transcription without completely abolishing acetyltransferase activity, whereas catalytic HAT inhibitors may produce broader systemic effects owing to global suppression of protein acetylation. Likewise, protein degraders may introduce additional safety concerns by eliminating both the catalytic and scaffolding functions of CBP/p300.

Beyond safety, another important consideration is the context-dependent biological role of CBP/p300. In addition to sustaining oncogenic transcriptional programs, CBP/p300 also function as transcriptional coactivators of wild-type p53 by promoting its acetylation and transcriptional activity. Therefore, the therapeutic response to CBP/p300 inhibitors may vary according to the TP53 mutation status, with tumors harboring mutant TP53 likely relying predominantly on suppression of enhancer-driven oncogenic transcription. Future clinical studies should incorporate TP53 status together with subtype-specific biomarkers to improve patient stratification and optimize therapeutic benefits.

Moreover, breast cancer patients frequently receive multimodal treatments, including anthracycline-based chemotherapy and long-term endocrine therapy, which may alter their cardiopulmonary function, bone marrow reserve, and metabolic status. Therefore, the direct extrapolation of safety data from other malignancies to breast cancer should be approached with caution. Future research should prioritize breast cancer-specific clinical studies incorporating pharmacokinetic analyses, biomarker-guided patient selection, and comprehensive long-term toxicity monitoring to better define the optimal therapeutic window for CBP/p300-targeted therapies.

## Figures and Tables

**Table 1:** Comparative characteristics of different CBP/p300 targeting modes.

Modality Type	Representative Molecule	Mechanism of Action	Main Advantages	Main Limitations	Potential Application Scenarios	Experimental Model and Level
BRD inhibitor	SGC-CBP30, GNE-049, I-CBP112	Competitively blocks the binding of the BRD to acetylated lysine	Enhancer-selective inhibition with relatively minor side effects	Inhibition is incomplete and may be compensated by other BRD proteins.	ER^+^ breast cancer (dependent on enhancers) [33,41]	In vitro and in vivo
HAT inhibitor	A-485, C646, L002	Competitively blocks acetyl-CoA binding, thereby globally inhibiting acetyltransferase activity	Exhibits strong inhibitory effects and can terminate the complete carcinogenic transcription program	A global decrease in acetylation may lead to normal cytotoxicity; poor pharmacokinetic properties	ER-positive breast cancer (particularly endocrine-resistant) [33]; part TNBC [47]	In vitro and in vivo
PROTAC degradant	dCBP-1, JQAD1, A8	Induces CBP/p300 ubiquitination and proteasomal degradation	Completely eradicates the target protein, overcomes partial drug resistance, and ensures prolonged efficacy	High molecular weight with low oral bioavailability; potential off-target degradation in normal tissues	Drug-resistant breast cancer; requiring long-term suppression of recurrent/metastatic lesions [50,51]	In vitro and in vivo
Dual-target inhibitor	NEO2734, NEO1132	Suppresses both the CBP/p300 BRD and the BET BRD simultaneously	Collaboratively inhibits transcription driven by super-enhancers, resulting in enhanced antitumor activity	Double inhibition may increase toxicity; evidence for breast cancer is limited	Breast cancer subtypes that are highly dependent on super-enhancers [23,53]	In vitro and in vivo

**Table 2:** Major combination therapy strategies for CBP/p300 inhibitors.

Joint Strategy	Molecular Basis	Target Population/Subtype	Experimental Model and Level
+Endocrine therapy (Tamoxifen/Frovixostatin)	Dual blockade of the ER transcriptional axis	ER-positive (particularly endocrine-resistant) [135]	In vitro
+Chemotherapy (paclitaxel/anthracyclines)	Downregulation of ABC transporter; inhibition of DNA repair; restriction of plasticity	TNBC (drug-resistant/high ABC expression) [136]	In vitro
+Anti-PD-1/PD-L1 immunotherapy	Reduces TANs; increases CD8^+^ T cells; upregulates MHC-I	TNBC (neutrophil-enriched/immunosuppressive type) [138,139]	In vitro and in vivo
+CDK4/6 inhibitor	Synergistic inhibition of the cell cycle	ER-positive (may extend to other subtypes) [141]	In vitro and in vivo
+PARP inhibitor	Inhibits DNA repair and induces synthetic lethal effects	BRCA mutation/homologous recombination deficiency type [143]	In vitro

**Table 3:** Clinically tested CBP/p300-targeting molecules and their translational significance in breast cancer.

Candidate Drug	Target/Mechanism of Action	Clinical Stage	Main Indications (Reported)	Specific Evidence for Breast Cancer	Insights and Limitations Regarding Breast Cancer Transformation	Experimental Model and Level
CCS1477	p300/CBP BRD inhibitor	Stage I/IIa; NHL has progressed to Stage II	mCRPC, metastatic breast cancer, and non-small cell lung cancer	Included in mBC, but without independent cohort data; the mechanism involves targeting AR and cMyc	Can be used for exploration of the AR-positive TNBC/HER2-positive/AR-positive subtype [146]; requires correlation with AR expression stratification	In vitro and in vivo
FT-7051	p300/CBP BRD inhibitor	Stage I (COURAGE) and Stages 1b/2a	mCRPC	No breast cancer enrollment records; AR pathway-oriented	Direct transformation candidate molecules for AR-positive breast cancer [19]; requires supporting biomarker strategies	In vitro and in vivo
TT125-802	p300/CBP BRD inhibitor	Stage I	NSCLC	No breast cancer data available	The first active signal for solid tumors [18]; safety advantages may favor combination therapy [20]; urgent need for a basket trial for breast cancer [148]	In vitro and in vivo
NEO2734	p300/CBP + BET dual BRD inhibitors	Stage I	mCRPC, NUT, CMML	No breast cancer cases were enrolled; downregulation of the MYC target gene pathway was identified as the mechanism	Breast cancer subtypes with high dependence on super-enhancers hold theoretical significance [54]; the dual inhibition toxicity requires evaluation [23]	In vitro and in vivo
Selective CBP degradation agent	CBP PROTAC degradation agent	In non-GLP toxicology studies	EP300-mutant tumor; ER-positive BC	Specifically targeted at ER^+^ breast cancer and EP300-mutated tumors; IND-ready by 2026	The degradation strategy achieves complete target clearance and may overcome partial drug resistance [50]; the ER/FOXA1 biomarker dependency requires validation	In vitro

## Data Availability

No new data were created or analyzed in this study. Data sharing is not applicable to this article.

## References

[1] Bray F., Laversanne M., Sung H., Ferlay J., Siegel R.L., Soerjomataram I., Jemal A. (2024). Global cancer statistics 2022: Globocan estimates of incidence and mortality worldwide for 36 cancers in 185 countries. CA Cancer J. Clin..

[2] Filho A.M., Laversanne M., Ferlay J., Colombet M., Piñeros M., Znaor A., Parkin D.M., Soerjomataram I., Bray F. (2024). The globocan 2022 cancer estimates: Data sources, methods, and a snapshot of the cancer burden worldwide. Int. J. Cancer.

[3] Pedersen R.N., Esen B.Ö., Mellemkjær L., Christiansen P., Ejlertsen B., Lash T.L., Nørgaard M., Cronin-Fenton D. (2021). The incidence of breast cancer recurrence 10–32 years after primary diagnosis. J. Natl. Cancer Inst..

[4] Yersal O., Barutca S. (2014). Biological subtypes of breast cancer: Prognostic and therapeutic implications. World J. Clin. Oncol..

[5] Newman L.A., Reis-Filho J.S., Morrow M., Carey L.A., King T.A. (2015). The 2014 society of surgical oncology Susan G. Komen for the cure symposium: Triple-negative breast cancer. Ann. Surg. Oncol..

[6] Łukasiewicz S., Czeczelewski M., Forma A., Baj J., Sitarz R., Stanisławek A. (2021). Breast cancer—Epidemiology, risk factors, classification, prognostic markers, and current treatment strategies—An updated review. Cancers.

[7] Saha S., Mahapatra S., Khanra S., Mishra B., Swain B., Malhotra D., Saha S., Panda V.K., Kumari K., Jena S. (2025). Decoding breast cancer treatment resistance through genetic, epigenetic, and immune-regulatory mechanisms: From molecular insights to translational perspectives. Cancer Drug Resist..

[8] Leonita A., Cheng S.Y., Warrick J., Kim I.Y., Deng S., Mu P. (2026). Game of clones: Decipher lineage plasticity in hormone-driven cancers. Cell. Mol. Life Sci..

[9] Shuai Y.J., Huang H.J. (2026). Transcriptional and epigenetic reprogramming, lineage plasticity and therapy resistance in prostate cancer. J. Natl. Cancer Cent..

[10] Ravindran S., Vini R., Rajavelu A., Harikumar K.B., Sreeja S. (2025). Navigating the complexities of epigenetic dysregulation in breast cancer and its implication in therapeutic interventions: A comprehensive overview. Mol. Cell. Biochem..

[11] Aristorena-Arruabarrena A., Toska E. (2022). Epigenetic mechanisms influencing therapeutic response in breast cancer. Front. Oncol..

[12] Ramadan W.S., Azawi A.M.A., Lozon L., Kawaf R.R., Zein R.A., Gharib Y.E., Awady R.E. (2025). Epigenetic therapeutics: Reprogramming triple-negative breast cancer into responsive subtypes. Endocr. Relat. Cancer.

[13] Zhou L.L., Yu C.W. (2024). Epigenetic modulations in triple-negative breast cancer: Therapeutic implications for tumor microenvironment. Pharmacol. Res..

[14] Davies M., Boyce M., Conway E. (2024). Short circuit: Transcription factor addiction as a growing vulnerability in cancer. Curr. Opin. Struct. Biol..

[15] Yazıcı E., McIntyre J. (2025). The complex network of p300/CBP regulation: Interactions, post-translational modifications, and therapeutic implications. J. Biol. Chem..

[16] Xiao L.Y., Jin H., Dang Y.N., Zhao P.P., Li S., Shi Y., Wang S.H., Zhang K. (2025). DUX-mediated configuration of p300/CBP drives minor zygotic genome activation independent of its catalytic activity. Cell Rep..

[17] Chen Q.J., Yang B.H., Liu X.C., Zhang X.D., Zhang L.R., Liu T. (2022). Histone acetyltransferases CBP/p300 in tumorigenesis and CBP/p300 inhibitors as promising novel anticancer agents. Theranostics.

[18] Masci D., Puxeddu M., Silvestri R., Regina G.L. (2024). Targeting CBP and p300: Emerging anticancer agents. Molecules.

[19] Xu H.T., Hou Y.F., Zhao Z.H., Zhang J.H., Li P., Cao Y.J., Nie X.B., Hou J.Q. (2025). CBP/p300, a promising therapeutic target for prostate cancer. J. Transl. Med..

[20] Wu X., Zhang X., Tang S.S., Wang Y. (2025). The important role of the histone acetyltransferases p300/CBP in cancer and the promising anticancer effects of p300/CBP inhibitors. Cell Biol. Toxicol..

[21] Waddell A.R., Huang H.J., Liao D.Q. (2021). CBP/p300: Critical co-activators for nuclear steroid hormone receptors and emerging therapeutic targets in prostate and breast cancers. Cancers.

[22] Yuan X.Y., Hao X.X., Chan H.L., Zhao N., Pedroza D.A., Liu F.S., Le K., Smith A.J., Calderon S.J., Lieu N. (2024). CREB-binding protein/P300 bromodomain inhibition reduces neutrophil accumulation and activates antitumor immunity in triple-negative breast cancer. Jci Insight.

[23] Strachowska M., Robaszkiewicz A. (2024). Characteristics of anticancer activity of CBP/p300 inhibitors—Features of their classes, intracellular targets and future perspectives of their application in cancer treatment. Pharmacol. Ther..

[24] Xu L.X., Xuan H.W., Shi X.B. (2025). Dysregulation of the p300/CBP histone acetyltransferases in human cancer. Epigenomics.

[25] Gou P.H., Zhang W.C. (2024). Protein lysine acetyltransferase CBP/p300: A promising target for small molecules in cancer treatment. Biomed. Pharmacother..

[26] Bacabac M., Xu W. (2023). Oncogenic super-enhancers in cancer: Mechanisms and therapeutic targets. Cancer Metastasis Rev..

[27] Narita T., Ito S., Higashijima Y., Chu W.K., Neumann K., Walter J., Satpathy S., Liebner T., Hamilton W.B., Maskey E. (2021). Enhancers are activated by p300/CBP activity-dependent PIC assembly, RNAPII recruitment, and pause release. Mol. Cell.

[28] Fan S., Gao Y.H., Dai X.Y., Ma H., Yang Z.C. (2025). Super enhancers as drivers of hallmarks of cancer: From oncogene activation to metastatic progression. Crit. Rev. Oncol. Hematol..

[29] Caligiuri M., Williams G.L., Castro J., Battalagine L., Wilker E., Yao L.L., Schiller S., Toms A., Li P., Pardo E. (2023). FT-6876, a potent and selective inhibitor of CBP/p300, is active in preclinical models of androgen receptor-positive breast cancer. Target. Oncol..

[30] Song X.Z., Zhang C.W., Zhao M.K., Chen H., Liu X., Chen J.W., Lonard D.M., Qin L., Xu J.M., Wang X.S. (2015). Steroid receptor Coactivator-3 (SRC-3/AIB1) as a novel therapeutic target in triple negative breast cancer and its inhibition with a phospho-bufalin prodrug. PLoS ONE.

[31] Zwart W., Theodorou V., Kok M., Canisius S., Linn S., Carroll J.S. (2011). Erratum to: Oestrogen receptor-co-factor-chromatin specificity in the transcriptional regulation of breast cancer. EMBO J..

[32] Chen L.Y., Roy S.J.S., Jadhav A.M., Wang W.W., Chen P.H., Bishop T., Erb M.A., Parker C.G. (2024). Functional investigations of p53 acetylation enabled by heterobifunctional molecules. ACS Chem. Biol..

[33] Waddell A., Mahmud I., Ding H.C., Huo Z.G., Liao D.Q. (2021). Pharmacological inhibition of CBP/p300 blocks estrogen receptor alpha (ERα) function through suppressing enhancer H3K27 acetylation in luminal breast cancer. Cancers.

[34] Krošel M., Gabathuler M., Moser L., Maciukiewicz M., Züllig T., Seifritz T., Tomšič M., Distler O., Ospelt C., Klein K. (2023). The histone acetyl transferases CBP and p300 regulate stress response pathways in synovial fibroblasts at transcriptional and functional levels. Sci. Rep..

[35] Cochran A.G., Conery A.R., Sims R.J. (2019). Bromodomains: A new target class for drug development. Nat. Rev. Drug Discov..

[36] Park S., Stanfield R.L., Martinez-Yamout M.A., Dyson H.J., Wilson I.A., Wright P.E. (2017). Role of the CBP catalytic core in intramolecular SUMOylation and control of histone H3 acetylation. Proc. Natl. Acad. Sci. USA.

[37] Hay D.A., Fedorov O., Martin S., Singleton D.C., Tallant C., Wells C., Picaud S., Philpott M., Monteiro O.P., Rogers C.M. (2014). Discovery and optimization of small-molecule ligands for the CBP/p300 bromodomains. J. Am. Chem. Soc..

[38] Raisner R., Kharbanda S., Jin L.Y., Jeng E., Chan E., Merchant M., Haverty P.M., Bainer R., Cheung T., Arnott D. (2018). Enhancer activity requires CBP/P300 bromodomain-dependent histone H3K27 acetylation. Cell Rep..

[39] Picaud S., Fedorov O., Thanasopoulou A., Leonards K., Jones K., Meier J., Olzscha H., Monteiro O., Martin S., Philpott M. (2015). Generation of a selective small molecule inhibitor of the CBP/p300 bromodomain for leukemia therapy. Cancer Res..

[40] Nagata A., Chiang E.Y., Jhunjhunwala S., Caplazi P., Arumugam V., Modrusan Z., Chan E., Merchant M., Jin L., Arnott D. (2019). Regulation of tumor-associated myeloid cell activity by CBP/EP300 bromodomain modulation of H3K27 acetylation. Cell Rep..

[41] Gao Y.J., Chen L.J., Han Y.L., Wu F.R., Yang W.-S., Zhang Z., Huo T., Zhu Y.M., Yu C.T., Kim H. (2020). Acetylation of histone H3K27 signals the transcriptional elongation for estrogen receptor alpha. Commun. Biol..

[42] Manickavinayaham S., Vélez-Cruz R., Biswas A.K., Bedford E., Klein B.J., Kutateladze T.G., Liu B., Bedford M.T., Johnson D.G. (2019). E2F1 acetylation directs p300/CBP-mediated histone acetylation at DNA double-strand breaks to facilitate repair. Nat. Commun..

[43] Lasko L.M., Jakob C.G., Edalji R.P., Qiu W., Montgomery D., Digiammarino E.L., Hansen T.M., Risi R.M., Frey R., Manaves V. (2017). Discovery of a selective catalytic p300/CBP inhibitor that targets lineage-specific tumours. Nature.

[44] Zhang L.L., Sheng C.X., Zhou F.Y., Zhu K.C., Wang S.S., Liu Q.Q., Yuan M.M., Xu Z.Q., Liu Y., Lu J.L. (2021). CBP/p300 HAT maintains the gene network critical for β cell identity and functional maturity. Cell Death Dis..

[45] Reed S.M., Quelle D.E. (2014). p53 acetylation: Regulation and consequences. Cancers.

[46] Shendy N.A.M., Bikowitz M., Sigua L.H., Zhang Y., Mercier A., Khashana Y., Nance S., Liu Q., Delahunty I.M., Robinson S. (2024). Group 3 medulloblastoma transcriptional networks collapse under domain specific EP300/CBP inhibition. Nat. Commun..

[47] Yang H., Pinello C.E., Luo J., Li D.W., Wang Y.F., Zhao L.Y., Jahn S.C., Saldanha S.A., Chase P., Planck J. (2013). Small-molecule inhibitors of acetyltransferase p300 identified by high-throughput screening are potent anticancer agents. Mol. Cancer Ther..

[48] Palaferri L., Cheng I., Nevado C. (2025). Recent advances in CBP/EP300 degraders. Chimia.

[49] Ojeda S., Vannam R., Sayilgan J., Karakyriakou B., Hu E., Kreuzer J., Morris R., Lopez-Xcanda I.H., Rai S., Haas W. (2021). Targeted degradation of the enhancer lysine acetyltransferases CBP and p300. Cell Chem. Biol..

[50] Lei Y.H., Tang Q., Ni Y., Li C.H., Luo P., Huang K., Chen X., Zhu Y.X., Wang N.Y. (2024). Design, synthesis and biological evaluation of new RNF126-based p300/CBP degraders. Bioorg. Chem..

[51] Tiwari P.K., Harrison D.A., Ojeda S., Kawale A.S., Doda S.R., Vannam R., Karakyriakou B., Koglin A.S., Rizvi S., Ott C.J. (2024). Activity of orally available CBP/p300 fegraders in pre-clinical models of multiple myeloma. Blood.

[52] Ma M.J., Li M.Y., Zhang C.W., Yang Z.X., Chen X.Y., Lu P.H., Nie S.S., Zhang S.Q., Ma S.M., Qin C. (2024). Discovery of a highly potent PROTAC degrader of p300/CBP proteins for the treatment of enzalutamide-resistant prostate cancer. J. Med. Chem..

[53] Qian H.H., Zhu M., Tan X.Y., Zhang Y.X., Liu X.N., Yang L. (2023). Super-enhancers and the super-enhancer reader BRD4: Tumorigenic factors and therapeutic targets. Cell Death Discov..

[54] Spriano F., Gaudio E., Cascione L., Tarantelli C., Melle F., Motta G., Priebe V., Rinaldi A., Golino G., Mensah A.A. (2020). Antitumor activity of the dual BET and CBP/EP300 inhibitor NEO2734. Blood Adv..

[55] Ryan K.R., Giles F., Morgan G.J. (2020). Targeting both BET and CBP/EP300 proteins with the novel dual inhibitors NEO2734 and NEO1132 leads to anti-tumor activity in Multiple Myeloma. Eur. J. Haematol..

[56] Choo N., Keerthikumar S., Ramm S., Ashikari D., Teng L.D., Niranjan B., Hedwards S., Porter L.H., Goode D.L., Simpson K.J. (2024). Co-targeting BET, CBP, and p300 inhibits neuroendocrine signalling in androgen receptor-null prostate cancer. J. Pathol..

[57] Giles F., Witcher M., Brown B. (2018). NEO2734: A novel potent oral dual BET and P300/CBP inhibitor. Ann. Oncol..

[58] Romero F.A., Murray J., Lai K.W., Tsui V., Albrecht B.K., An L., Beresini M.H., de-Leon B.G., Bronner S.M., Chan E.W. (2017). Gne-781, a highly advanced potent and selective bromodomain inhibitor of cyclic adenosine monophosphate response element binding protein, binding protein (CBP). J. Med. Chem..

[59] Whedon S.D., Cole P.A. (2022). KATs off: Biomedical insights from lysine acetyltransferase inhibitors. Curr. Opin. Chem. Biol..

[60] Jaschke N.P., Breining D., Hofmann M., Pählig S., Baschant U., Oertel R., Traikov S., Grinenko T., Saettini F., Biondi A. (2024). Small-molecule CBP/p300 histone acetyltransferase inhibition mobilizes leukocytes from the bone marrow via the endocrine stress response. Immunity.

[61] Durbin A.D., Wang T.J., Wimalasena V.K., Zimmerman M.W., Li D., Dharia N.V., Mariani L., Shendy N.A.M., Nance S., Patel A.G. (2021). EP300 selectively controls the enhancer landscape of MYCN-amplified neuroblastoma. Cancer Discov..

[62] Kasper L.H., Fukuyama T., Biesen M.A., Boussouar F., Tong C., de-Pauw A., Murray P.J., van-Deursen J.M.A., Brindle P.K. (2006). Conditional knockout mice reveal distinct functions for the global transcriptional coactivators CBP and p300 in T-cell development. Mol. Cell. Biol..

[63] Partanen A., Motoyama J., Hui C.C. (1999). Developmentally regulated expression of the transcriptional cofactors/histone acetyltransferases CBP and p300 during mouse embryogenesis. Int. J. Dev. Biol..

[64] Wang S.S., Li T.J., Sheng C.X., Tan J.L., Yang Y.L., Ma X.Q., Liu Y., Wei R., Zhou F.Y., Zhou L.B. (2026). CBP/p300 is critical for the expansion and maintenance of functional pancreatic α cell mass. Nat. Commun..

[65] Armstrong A.J., Gordon M.S., Reimers M.A., Sedkov A., Lipford K., Snavely M.J., Kumar S., Guichard S.M., Shore N. (2021). The Courage study: A first-in-human phase 1 study of the CBP/p300 inhibitor FT-7051 in men with metastatic castration-resistant prostate cancer. J. Clin. Oncol..

[66] Murakami S., Nagari A., Kraus W.L. (2017). Dynamic assembly and activation of estrogen receptor α enhancers through coregulator switching. Genes Dev..

[67] Kong S.L., Li G.L., Loh S.L., Sung W.-K., Liu E.T. (2011). Cellular reprogramming by the conjoint action of ERα, FOXA1, and GATA3 to a ligand-inducible growth state. Mol. Syst. Biol..

[68] Pavithran H., Kumavath R. (2021). Emerging role of pioneer transcription factors in targeted ERα positive breast cancer. Explor. Target. Anti-Tum. Ther..

[69] Bose D.A., Donahue G., Reinberg D., Shiekhattar R., Bonasio R., Berger S.L. (2017). RNA binding to CBP stimulates histone acetylation and transcription. Cell.

[70] Li Z.Q., Wu Y., Yates M.E., Tasdemir N., Bahreini A., Chen J., Levine K.M., Priedigkeit N.M., Nasrazadani A., Ali S. (2022). Hotspot ESR1 mutations are multimodal and contextual modulators of breast cancer metastasis. Cancer Res..

[71] Martin L.A., Ribas R., Simigdala N., Schuster E., Pancholi S., Tenev T., Gellert P., Buluwela L., Harrod A., Thornhill A. (2017). Discovery of naturally occurring ESR1 mutations in breast cancer cell lines modelling endocrine resistance. Nat. Commun..

[72] Gates L.A., Gu G.W., Chen Y., Rohira A.D., Lei J.T., Hamilton R.A., Yu Y., Lonard D.M., Wang J., Wang S.-P. (2018). Proteomic profiling identifies key coactivators utilized by mutant ERα proteins as potential new therapeutic targets. Oncogene.

[73] GarciaMartinez L., Zhang Y.S., Nakata Y., Chan H.L., Morey L. (2021). Epigenetic mechanisms in breast cancer therapy and resistance. Nat. Commun..

[74] Basho R.K., Zhao L., White J.B., Huo L., Bassett R.L., Mittendorf E.A., Thompson A., Litton J.K., Ueno N., Arun B. (2024). Comprehensive analysis identifies variability in PI3K pathway alterations in triple-negative breast cancer subtypes. JCO Precis. Oncol..

[75] Lee M., Yoo T.K., Chae B.J., Lee A., Cha Y.J., Lee J., Ahn S.G., Kang J. (2025). Author correction: Luminal androgen receptor subtype and tumor-infiltrating lymphocytes groups based on triple-negative breast cancer molecular subclassification. Sci. Rep..

[76] Taifour T., Attalla S.S., Zuo D., Gu Y., SanguinGendreau V., Proud H., Solymoss E., Bui T., Kuasne H., Papavasiliou V. (2023). The tumor-derived cytokine Chi3l1 induces neutrophil extracellular traps that promote T cell exclusion in triple-negative breast cancer. Immunity.

[77] Qutami F.A., AlHalabi W., Vijayakumar A., Rawat S.S., Mossa A.H., Jayakumar M.N., Samreen B., Hachim M.Y. (2024). Characterizing the inflammatory profile of neutrophil-rich triple-negative breast cancer. Cancers.

[78] Gabrilovich D.I., Nagaraj S. (2009). Myeloid-derived suppressor cells as regulators of the immune system. Nat. Rev. Immunol..

[79] Liu J.H., Wang X.Y., He D.H., Maasoumyhaghighi H., Nouri M., Wu M., Peng J., Rao X.J., Wang R.X., Wu S. (2025). Therapeutic targeting of the p300/CBP bromodomain enhances the efficacy of immune checkpoint blockade therapy. Oncogene.

[80] Xu F.F., Sun H.M., Fang R.P., Zhang L., Shi H., Wang X., Fu X.L., Li X.M., Shi X.H., Wu Y. (2021). The modulation of PD-L1 induced by the oncogenic HBXIP for breast cancer growth. Acta Pharmacol. Sin..

[81] Wang H.N., Yi X.L., Wang X.X., Yang Y.Q., Zhang H.X., Wang H., Chen J.R., Zhang B.L., Guo S., Wu L.L. (2024). Nucleo-cytosolic acetyl-CoA drives tumor immune evasion by regulating PD-L1 in melanoma. Cell Rep..

[82] Strachowska M., Gronkowska K., Michlewska S., Robaszkiewicz A. (2021). CBP/p300 bromodomain inhibitor-I-CBP112 declines transcription of the key ABC transporters and sensitizes cancer cells to chemotherapy drugs. Cancers.

[83] Lai R.Z., Lin Z.Q., Yang C.Y., Hai L., Yang Z.Z., Guo L., Nie R.F., Wu Y. (2024). Novel berberine derivatives as p300 histone acetyltransferase inhibitors in combination treatment for breast cancer. Eur. J. Med. Chem..

[84] Luo J., Chen Z.X., Qiao Y.Y., Tien J.C.Y., Young E., Mannan R., Mahapatra S., Bhattacharyya R., Xiao L.B., He T.C. (2025). Targeting histone H2B acetylated enhanceosomes via p300/CBP degradation in prostate cancer. Nat. Genet..

[85] Shome R., Samur M., Talluri S., Matsubara R., Joshi A., Rexha F., Cirstea D., Yee A., Munshi N., Raje N. (2025). Deciphering the role of EP300 in maintaining the enhancer landscape and lineage-specific epigenetic vulnerability in multiple myeloma. Blood.

[86] Shah V., Giotopoulos G., Osaki H., Meyerhöfer M., Meduri E., Crespo A.G., Behrendt M.A., Pañella M.S., Tarkar A., Schubert B. (2024). Acute resistance to BET inhibitors remodels compensatory transcriptional programs via p300 coactivation. Blood.

[87] Chi Y.Y., Xue J.Y., Huang S., Xiu B.Q., Su Y.H., Wang W., Guo R., Wang L., Li L., Shao Z.M. (2019). CapG promotes resistance to paclitaxel in breast cancer through transactivation of PIK3R1/P50. Theranostics.

[88] Strachowska M., Gronkowska K., Sobczak M., Grodzicka M., Michlewska S., Kołacz K., Sarkar T., Korszun J., Ionov M., Robaszkiewicz A. (2023). I-CBP112 declines overexpression of ATP-binding cassette transporters and sensitized drug-resistant MDA-MB-231 and A549 cell lines to chemotherapy drugs. Biomed. Pharmacother..

[89] Schlam I., Tarantino P., Tolaney S.M. (2022). Overcoming resistance to HER2-directed therapies in breast cancer. Cancers.

[90] Duan N.J., Hua Y.J., Yan X.Q., He Y.Z., Zeng T.Y., Gong J., Fu Z.Y., Li W., Yin Y.M. (2024). Unveiling alterations of epigenetic modifications and chromatin architecture leading to lipid metabolic reprogramming during the evolutionary trastuzumab adaptation of HER2-positive breast cancer. Adv. Sci..

[91] Rojhannezhad M., Soltani B.M., Vasei M., Ghorbanmehr N., Mowla S.J. (2023). Functional analysis of a putative HER2-associated expressed enhancer, Her2-Enhancer1, in breast cancer cells. Sci. Rep..

[92] Zhao H.Y., Feng K., Lei J.J., Shu Y.P., Bo L., Liu Y., Wang L.X., Liu W.Y., Ning S.W., Wang L. (2024). Identification of somatic mutation-driven enhancers and their clinical utility in breast cancer. iScience.

[93] Hao X.Y., Zheng J.L., Yu X.Q., Li Z.X., Ren G.H. (2023). β-catenin promotes resistance to trastuzumab in breast cancer cells through enhancing interaction between HER2 and SRC. Acta Biochim. Pol..

[94] Higuchi Y., Teo J.L., Yi D., Kahn M. (2025). Safely targeting cancer, the wound that never heals, utilizing CBP/beta-catenin antagonists. Cancers.

[95] Takemaru K.I., Moon R.T. (2000). The transcriptional coactivator CBP interacts with β-catenin to activate gene expression. J. Cell Biol..

[96] Zhu M.Z., Lu X.J., Wang D.H., Ma J.H., Wang Y., Wang R., Wang H.Y., Cheng W.H., Zhu Y.L. (2025). A narrative review of epigenetic marker in H3K27ac and its emerging potential as a therapeutic target in cancer. Epigenomics.

[97] Xiao X.S., Cai M.Y., Chen J.W., Guan X.Y., Kung H.F., Zeng Y., Xie D. (2011). High Expression of p300 in Human Breast Cancer Correlates with Tumor Recurrence and Predicts Adverse Prognosis. Chin. J. Cancer Res..

[98] Gari M., Baothman B.K., Gari K., Alhomrani M., Alsehli H., Bagarish A.G., Hameed Y., Natto M., Alkhatabi H., Abuzenadah A. (2025). A combined transcriptomic, epigenetic, and functional analysis identifies novel biomarkers in breast cancer. Hereditas.

[99] Lusby R., Zhang Z.Y., Mahesh A., Tiwari V.K. (2024). Decoding gene regulatory circuitry underlying TNBC chemoresistance reveals biomarkers for therapy response and therapeutic targets. npj Precis. Oncol..

[100] Imran K., Iqbal M.J., Ahmed M.M., Khalid A., Cortés H., Hernández O.D.R., González G.F., Gómez G.L., Falzone L., Libra M. (2025). Epigenetic dysregulation in cancer: Mechanisms, diagnostic biomarkers and therapeutic strategies. Med. Oncol..

[101] Zeng Q.M., Wang K., Zhao Y.X., Ma Q.Z., Chen Z.N., Huang W. (2023). Effects of the acetyltransferase p300 on tumour regulation from the novel perspective of posttranslational protein modification. Biomolecules.

[102] Green A.R., Burney C., Granger C.J., Paish E.C., El-Sheikh S., Rakha E.A., Powe D.G., Macmillan R.D., Ellis I.O., Stylianou E. (2008). The prognostic significance of steroid receptor co-regulators in breast cancer: Co-repressor NCOR2/SMRT is an independent indicator of poor outcome. Breast Cancer Res. Treat..

[103] Garcia-Carpizo V., Ruiz-Llorente S., Sarmentero J., González-Corpas A., Barrero M.J. (2019). CREBBP/EP300 bromodomain inhibition affects the proliferation of AR-positive breast cancer cell lines. Mol. Cancer Res..

[104] Deng X.Y., Ma C.L., Chen X.Y., Yi M., Cao Q.H., Liao R.C., Lei X.Y., Bai L.C., Zhao B., Wang Y.N. (2025). Acetylation suppresses breast cancer progression by sustaining CLYBL stability. J. Transl. Med..

[105] Liu X., Gillis N., Jiang C., McCofie A., Shaw T.I., Tan A.C., Zhao B., Wan L.X., Duckett D.R., Teng M.X. (2024). An Epigenomic fingerprint of human cancers by landscape interrogation of super enhancers at the constituent level. PLoS Comput. Biol..

[106] Valenciaga A., Saji M., Yu L.B., Zhang X.L., Bumrah C., Yilmaz A.S., Knippler C.M., Miles W., Giordano T.J., Cote G.J. (2018). Transcriptional targeting of oncogene addiction in medullary thyroid cancer. J. Clin. Investig..

[107] Luo Z.H., Shi M.W., Zhang Y., Wang D.Y., Tong Y.B., Pan X.L., Cheng S.S. (2023). CenhANCER: A comprehensive cancer enhancer database for primary tissues and cell lines. Database.

[108] BommiReddy A., ParkChouinard S., Mayhew D.N., Terzo E., Hingway A., Steinbaugh M.J., Wilson J.E., Sims R.J., Conery A.R. (2022). CREBBP/EP300 acetyltransferase inhibition disrupts FOXA1-bound enhancers to inhibit the proliferation of ER+ breast cancer cells. PLoS ONE.

[109] Chen Q.S., Cai R.Z., Wang Y., Liang G.H., Zhang K.M., Yang X.Y., Yang D., Zhao D.C., Zhu X.F., Deng R. (2025). Profiling triple-negative breast cancer-specific super-enhancers identifies high-risk mesenchymal development subtype and BETi-Targetable vulnerabilities. Mol. Cancer.

[110] Zawistowski J.S., Bevill S.M., Goulet D.R., Stuhlmiller T.J., Beltran A.S., Olivares-Quintero J.F., Singh D., Sciaky N., Parker J.S., Rashid N.U. (2017). Enhancer remodeling during adaptive bypass to MEK inhibition is attenuated by pharmacologic targeting of the P-TEFb complex. Cancer Discov..

[111] Lin L.Y., Li H.M., Wang X., Wang Z.Z., Su G.H., Zhou J.Y., Sun S.Y., Ma X.W., Chen Y., You C. (2023). Components of the tumor immune microenvironment based on m-IHC correlate with prognosis and subtype of triple-negative breast cancer. Cancer Med..

[112] Kim U., Debnath R., Maiz J.E., Rico J., Sinha S., Blanco M.A., Chakrabarti R. (2024). ΔNp63 regulates MDSC survival and metabolism in triple-negative breast cancer. iScience.

[113] Gallagher S.J., Shklovskaya E., Hersey P. (2017). Epigenetic modulation in cancer immunotherapy. Curr. Opin. Pharmacol..

[114] Gazinska P., Milton C., Iacovacci J., Ward J., Buus R., Alaguthurai T., Graham R., Akarca A., Lips E., Naidoo K. (2022). Dynamic changes in the NK-, neutrophil-, and B-cell immunophenotypes relevant in high metastatic risk post neoadjuvant chemotherapy-resistant early breast cancers. Clin. Cancer Res..

[115] Lee J.V., Housley F., Yau C., Nakagawa R., Winkler J., Anttila J.M., Munne P.M., Savelius M., Houlahan K.E., Van-de-Mark D. (2022). Author Correction: Combinatorial immunotherapies overcome MYC-driven immune evasion in triple negative breast cancer. Nat. Commun..

[116] Taylor B.C., Sun X.P., GonzalezEricsson P.I., Sanchez V., Sanders M.E., Wescott E.C., Opalenik S.R., Hanna A., Chou S., Van K.L. (2023). NKG2A is a therapeutic vulnerability in immunotherapy resistant MHC-I heterogeneous triple negative breast cancer. Cancer Discov..

[117] Zhang W., Zhai Y.H., Cai Y., Gong X., Jiang Y.X., Rong R., Zheng C., Zhu B.Y., Zhu H.H., Wang H. (2024). Enhancing immunotherapy efficacy against MHC-I deficient triple-negative breast cancer using LCL161-loaded macrophage membrane-decorated nanoparticles. Acta Pharm. Sin. B..

[118] Arranz-Nicolás J., Ogando J., Soutar D., Arcos-Pérez R., Meraviglia-Crivelli D., Mañes S., Mérida I., Ávila-Flores A. (2018). Diacylglycerol kinase α inactivation is an integral component of the costimulatory pathway that amplifies TCR signals. Cancer Immunol. Immunother..

[119] Heger L., Heidkamp G.F., Amon L., Nimmerjahn F., Bäuerle T., Maier A., Erber R., Hartmann A., Hack C.C., Ruebner M. (2024). Unbiased high-dimensional flow cytometry identified NK and DC immune cell signature in Luminal A-type and triple negative breast cancer. Oncoimmunology.

[120] Patysheva M.R., Iamshchikov P.S., Fedorenko A.A., Bragina O.D., Vostrikova M.A., Garbukov E.Y., Cherdyntseva N.V., Denisov E.V., Gerashchenko T.S. (2025). Single-cell transcriptomic profiling of immune landscape in triple-negative breast cancer during neoadjuvant chemotherapy. npj Syst. Biol. Appl..

[121] Bishop T.R., Subramanian C., Bilotta E.M., GarnarWortzel L., Ramos A.R., Zhang Y.X., Asiaban J.N., Ott C.J., Rock C.O., Erb M.A. (2023). Acetyl-CoA biosynthesis drives resistance to histone acetyltransferase inhibition. Nat. Chem. Biol..

[122] Cremers C.G., Nguyen L.K. (2019). Network rewiring, adaptive resistance and combating strategies in breast cancer. Cancer Drug Resist..

[123] Wang K., Baird L., Yamamoto M. (2025). The clinical-grade CBP/p300 inhibitor CCS1477 represses the global NRF2-dependent cytoprotective transcription program and re-sensitizes cancer cells to chemotherapeutic drugs. Free Radic. Biol. Med..

[124] Wu D., Yan Y.Q., Wei T., Ye Z.Q., Xiao Y.T., Pan Y.Q., Orme J.J., Wang D.J., Wang L.G., Ren S.C. (2021). An acetyl-histone vulnerability in PI3K/AKT inhibition-resistant cancers is targetable by both BET and HDAC inhibitors. Cell Rep..

[125] Kahl M., Brioli A., Bens M., Perner F., Kresinsky A., Schnetzke U., Hinze A., Sbirkov Y., Stengel S., Simonetti G. (2020). Correction: The acetyltransferase GCN5 maintains ATRA-resistance in non-APL AML. Leukemia.

[126] Tzelepis K., Koike-Yusa H., Braekeleer E.D., Li Y.L., Metzakopian E., Dovey O.M., Mupo A., Grinkevich V., Li M., Mazan M. (2016). A CRISPR dropout screen identifies genetic vulnerabilities and therapeutic targets in acute myeloid leukemia. Cell Rep..

[127] Roe J.-S., Mercan F., Rivera K., Pappin D.J., Vakoc C.R. (2015). BET Bromodomain Inhibition Suppresses the Function of Hematopoietic Transcription Factors in Acute Myeloid Leukemia. Mol. Cell.

[128] Zhang X., Zegar T., Lucas A., Morrison-Smith C., Knox T., French C.A., Knapp S., Müller S., Siveke J.T. (2020). Therapeutic targeting of p300/CBP HAT domain for the treatment of NUT midline carcinoma. Oncogene.

[129] He K.J., Xu T., Xu Y.C., Ring A., Kahn M., Goldkorn A. (2014). Cancer cells acquire a drug resistant, highly tumorigenic, cancer stem-like phenotype through modulation of the PI3K/Akt/β-catenin/CBP pathway. Int. J. Cancer.

[130] Manegold P., Lai K.K.Y., Wu Y.F., Teo J.L., Lenz H.J., Genyk Y.S., Pandol S.J., Wu K., Lin D.P., Chen Y.B. (2018). Differentiation Therapy Targeting the β-Catenin/CBP Interaction in Pancreatic Cancer. Cancers.

[131] Miller K.D., Pniewski K., Perry C.E., Papp S.B., Shaffer J.D., VelascoSilva J.N., Casciano J.C., Aramburu T.M., Srikanth Y.V.V., Cassel J. (2021). Targeting ACSS2 with a Transition-State Mimetic Inhibits Triple-Negative Breast Cancer Growth. Cancer Res..

[132] Zhu R.X., Ye X.L., Lu X.T., Xiao L.W., Yuan M., Zhao H., Guo D., Meng Y., Han H.K., Luo S.D. (2024). ACSS2 acts as a lactyl-CoA synthetase and couples KAT2A to function as a lactyltransferase for histone lactylation and tumor immune evasion. Cell Metab..

[133] Wang Y.Y., Su K.Q., Wang C., Deng T., Liu X.F., Sun S.Q., Jiang Y., Zhang C.F., Xing B.C., Du X.J. (2024). Chemotherapy-induced acetylation of ACLY by NAT10 promotes its nuclear accumulation and acetyl-CoA production to drive chemoresistance in hepatocellular carcinoma. Cell Death Dis..

[134] Mogol A.N., Yoo J.Y., Arredondo-Eve A., Goel M., Dutton D.J., Schane C.P., Lam A., Dutta D., Barnick B., Erdogan E.D. (2025). ACSS2-Mediated Metabolic-Epigenetic Crosstalk Drives Fulvestrant Resistance and Represents a Novel Therapeutic Target. npj Breast Cancer.

[135] Xu B.H., Li Q., Chen N., Zhu C.X., Meng Q.R., Ayyanathan K., Qian W.L., Jia H., Wang J.M., Ni P.H. (2019). The LIM protein Ajuba recruits DBC1 and CBP/p300 to acetylate ERα and enhances ERα target gene expression in breast cancer cells. Nucleic Acids Res..

[136] Pao G.M., Janknecht R., Ruffner H., Hunter T., Verma I.M. (2000). CBP/p300 interact with and function as transcriptional coactivators of BRCA1. Proc. Natl. Acad. Sci. USA.

[137] Wang Y.B., Zhang T.H., Kwiatkowski N., Abraham B.J., Lee T.I., Xie S.Z., Yuzugullu H., Von T., Li H.Y., Lin Z. (2015). CDK7-Dependent Transcriptional Addiction in Triple-Negative Breast Cancer. Cell.

[138] Zhou Y.X., Bastian I.N., Long M.D., Dow M., Li W.H., Liu T., Ngu R.K., Antonucci L., Huang J.Y., Phung Q.T. (2021). Activation of NF-κB and p300/CBP potentiates cancer chemoimmunotherapy through induction of MHC-I antigen presentation. Proc. Natl. Acad. Sci. USA.

[139] Tang Y.J., Cui G.Z., Liu H.C., Han Y., Cai C.J., Feng Z.Y., Shen H., Zeng S. (2024). Converting “cold” to “hot”: Epigenetics strategies to improve immune therapy effect by regulating tumor-associated immune suppressive cells. Cancer Commun..

[140] Brighi N., Conteduca V., Lolli C., Gurioli G., Schepisi G., Palleschi M., Mariotti M., Casadei C., De-Giorgi U. (2021). Corrigendum to “The cyclin-dependent kinases pathway as a target for prostate cancer treatment: Rationale and future perspectives” [Crit. Rev. Oncol. Hematol. 157 (January) (2021) 103199]. Crit. Rev. Oncol. Hematol..

[141] Zhou Y.K., Jin X., Ma J., Ding D.L., Huang Z.L., Sheng H.Y., Yan Y.Q., Pan Y.Q., Wei T., Wang L.G. (2021). HDAC5 Loss Impairs RB Repression of Pro-Oncogenic Genes and Confers CDK4/6 Inhibitor Resistance in Cancer. Cancer Res..

[142] Sardar S., McNair C.M., Ravindranath L., Chand S.N., Yuan W., Bogdan D., Welti J., Sharp A., Ryan N.K., Knudsen L.A. (2024). AR coactivators, CBP/p300, are critical mediators of DNA repair in prostate cancer. Oncogene.

[143] Li X., Zou L. (2024). BRCAness, DNA gaps, and gain and loss of PARP inhibitor-induced synthetic lethality. J. Clin. Investig..

[144] Sellars E., Savguira M., Wu J., Cancelliere S., Jen M., Krishnan R., Hakem A., Lovejoy D.B., Hakem R., Narod S.A. (2024). A high-throughput approach to identify BRCA1-downregulating compounds to enhance PARP inhibitor sensitivity. iScience.

[145] Wang H., Song D.D., Miao M., Wu S.M., Wang J., Huang Z., Ding H. (2025). Medicinal chemistry approaches to the discovery and development of p300/CBP inhibitors for cancer therapy. Eur. J. Med. Chem..

[146] Welti J., Sharp A., Brooks N., Yuan W., McNair C., Chand S.N., Pal A., Figueiredo I., Riisnaes R., Gurel B. (2021). Targeting p300/CBP axis in lethal prostate cancer. Cancer Discov..

[147] He Z.X., Wei B.F., Zhang X., Gong Y.P., Ma L.Y., Zhao W. (2020). Current development of CBP/p300 inhibitors in the last decade. Eur. J. Med. Chem..

[148] Nicosia L., Spencer G.J., Brooks N., Amaral F.M.R., Basma N.J., Chadwick J.A., Revell B., Wingelhofer B., MaiquesDiaz A., Sinclair O. (2023). Therapeutic targeting of EP300/CBP by bromodomain inhibition in hematologic malignancies. Cancer Cell.

